# Central adiposity and subclinical cardiac remodeling in Kyrgyz adults

**DOI:** 10.1016/j.ahjo.2026.100992

**Published:** 2026-10-24

**Authors:** Kylychbek Suiunov, Andrey Polupanov, Kyialbek Sakibaev, Meerimgul Sherikbai kyzy, Zhazgul Imetova, Anara Zhanbaeva, Nazgul Tashmatova, Aigul Momunova, Lazokatkhan Dzhumaeva, Akpay S. Sarybaev, Akylbek Sydykov, Argen Mamazhakypov

**Affiliations:** a International Medical Faculty, Osh State University, Osh, Kyrgyzstan; b Akhunbaev Kyrgyz State Medical Academy, Bishkek, Kyrgyzstan; c Osh International Medical University, Osh, Kyrgyzstan; d Medical Center Cardio Asia Plus, Osh, Kyrgyzstan; e Osh State University Medical Clinic, Osh State University, Osh, Kyrgyzstan; f Center for Postgraduate and Continuing Medical Education, Osh State University, Osh, Kyrgyzstan; g National Center of Cardiology and Internal Medicine, Bishkek, Kyrgyzstan; h Kyrgyz-Indian Mountain Biomedical Research Center, Bishkek, 720040, Kyrgyzstan; i Justus Liebig University of Giessen, Giessen, Germany

**Keywords:** Obesity, Central obesity, Cardiac remodeling, Left ventricular hypertrophy, Waist circumference

## Abstract

**Objective:**

Central adiposity may be associated with cardiac remodeling, but this remains unstudied in the Kyrgyz population. We examined associations of general (body mass index (BMI)) and central (waist circumference (WC), waist-to-height ratio (WHtR)) adiposity with cardiac alterations in a selected cohort of Kyrgyz adults without established cardiovascular disease, diabetes, or hypertension.

**Methods:**

In this cross-sectional study, 100 adults (median age, 39.0 years; 54% women) underwent echocardiography. Associations were evaluated using partial Spearman correlations adjusted for age and systolic blood pressure, multivariable regression, sex-interaction testing, and adjusted trend analysis across WC quartiles.

**Results:**

BMI, WC, and WHtR showed FDR-significant partial correlations with seven structural parameters: LA diameter, LA volume, IVS thickness, LVPW thickness, LVEDD, LVESD, and LV mass. For these outcomes, partial correlation coefficients ranged from 0.332–0.602 for BMI, 0.443–0.636 for WC, and 0.314–0.549 for WHtR. In separate adjusted regression models, BMI, WC, and WHtR were each associated with all seven structural outcomes. Diastolic indices showed no FDR-significant adjusted associations, and no evidence of WC × sex interaction was observed. Adjusted trends occurred across WC quartiles for all structural parameters (all nominal P for trend <0.05), without deviation from linearity.

**Conclusion:**

Adiposity measures, including WC, were associated with several cardiac structural measures in this selected cohort. Structural remodeling coexisted with preserved systolic function and no FDR-significant adjusted associations for the diastolic indices. These findings require confirmation in larger prospective studies because of the small, single-center, urban, cross-sectional design (n = 100).

## Highlights

• WC showed positive adjusted associations with cardiac structural measures in Kyrgyz adults (n=100)
• Partial Spearman correlations assessed WC, BMI, and WHtR with cardiac structural parameters after adjustment for age and systolic BP
• WC showed linear adjusted trends across quartiles for structural cardiac parameters
• First echocardiographic study of obesity-related cardiac remodeling in Kyrgyz adult

## Graphical abstract

**Figure:**
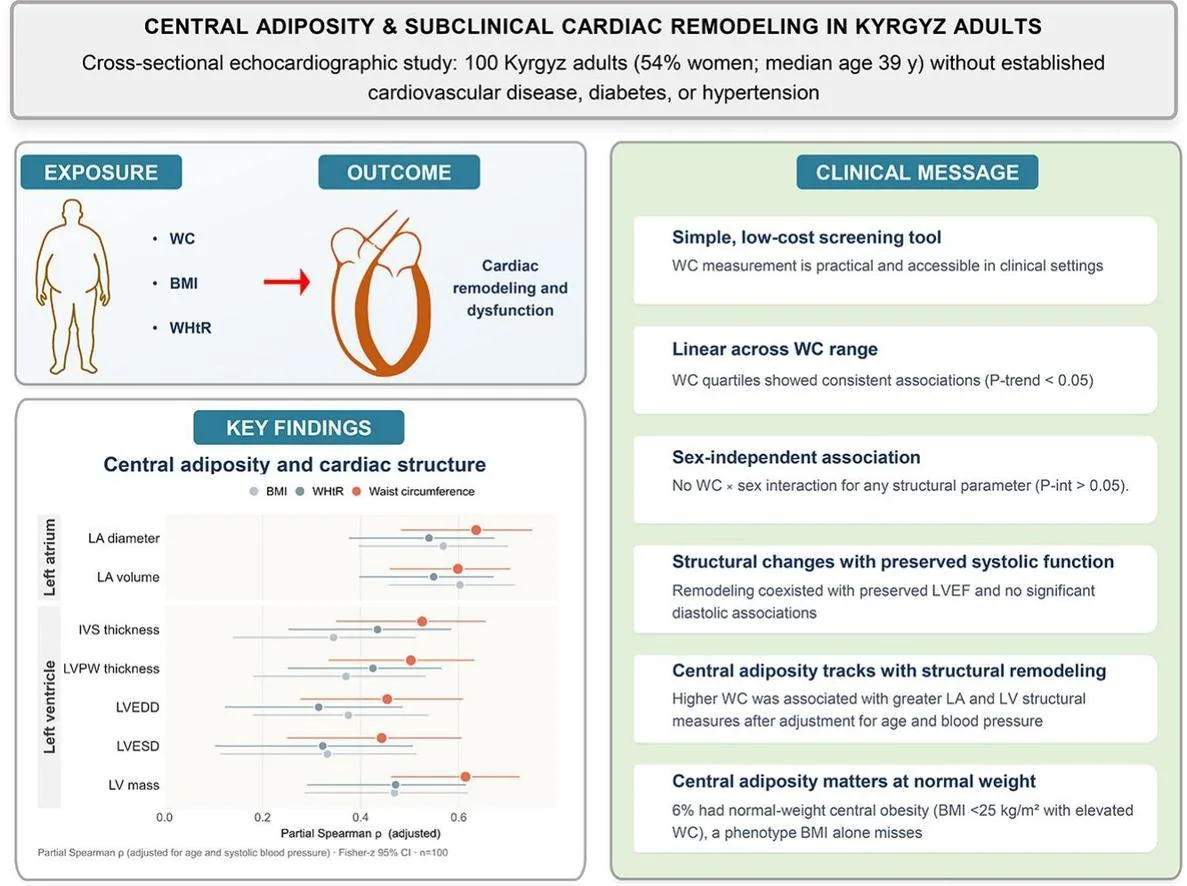

## 1 Introduction

Obesity is a pathological accumulation of adipose tissue that is becoming increasingly prevalent and is strongly associated with higher rates of cardiovascular disease, certain cancers, metabolic disorders, and chronic kidney disease [1], [2]. Analysis of adipose tissue distribution suggests that central adiposity, rather than general obesity defined by body mass index (BMI), more accurately reflects regional fat accumulation and exhibits stronger associations with cardiometabolic risk [3], [4], [5], [6], [7], [8], [9]. Moreover, central obesity shows stronger associations with cardiac remodeling and dysfunction than does general obesity [7]. Elevated BMI is associated with increased heart-failure risk; in a prospective meta-analysis, the nonlinear association with heart-failure incidence showed a threshold at approximately 23–24 kg/m^2^ [10]. Prospective and cross-sectional studies have also linked central adiposity measures, including waist circumference (WC), waist-to-hip ratio (WHR), waist-to-height ratio (WHtR) and visceral adipose tissue (VAT), with left ventricular (LV) mass, hypertrophy, and dilation [11], [12], [13], [14], [15], [16]. Furthermore, WC, WHR, and VAT are associated with adverse LV systolic [15], [17], [18], [19], [20] and diastolic function measures [11], [13], [14], [20], [21]. Kyrgyzstan and the broader Central Asian region carry a substantial and under-characterized cardiometabolic burden. For example, national data report 23.1% obesity prevalence [22], and age-standardized ischemic heart disease mortality in Central Asia was approximately 2.4-fold the global rate in 2021 [23], yet echocardiographic data on adiposity-related cardiac remodeling in this population remain limited. Most studies examining associations between adiposity and cardiac remodeling were conducted in Europe or in the United States and associations of general and central adiposity with cardiac remodeling and dysfunction in the Kyrgyz population remain unexplored. In this study, we aimed to assess associations of general adiposity (BMI) and central adiposity (WC, WHtR) with echocardiographic structural and functional parameters in participants without established cardiovascular disease. Secondary objectives were to test whether the association between waist circumference and cardiac structural parameters differed by sex and to characterize its adjusted trend across waist circumference quartiles.

## 2 Methods

### 2.1 General measures

A cross-sectional study was conducted at the outpatient cardiology clinic of Medical Center Cardio-Asia Plus, Osh, Kyrgyzstan, from September 1, 2024, to September 30, 2025. Participants were recruited through a voluntary, invitation-based preventive cardiovascular screening program targeting asymptomatic adults without cardiovascular complaints. The sample size was determined by the number of eligible participants recruited during the study period; no a priori sample-size calculation was performed. Adults aged 19–65 years who provided written informed consent were enrolled. Elevated WC was defined using sex-specific thresholds (≥102 cm in men and ≥88 cm in women) [24]. All participants had sinus rhythm on resting electrocardiography, no documented cardiovascular diagnosis, hospitalization, symptoms, or health complaints, and were not receiving cardiovascular medications at recruitment. Exclusion criteria included known cardiovascular disease, diabetes mellitus, documented hypertension or antihypertensive drug use, renal or hepatic dysfunction, thyroid disease, and any clinically significant abnormal baseline findings.

We measured body weight using a calibrated digital column scale and standing height using a wall-mounted stadiometer. BMI was calculated as weight in kilograms divided by height in meters squared. WC was measured at the upper border of the iliac crest at end expiration. Body surface area (BSA) was computed using the Du Bois formula: BSA (m^2^) = 0.007184 × (height in centimeters)^0.725^ × (weight in kilograms)^0.425^. After 5 min of seated rest, systolic and diastolic blood pressures and resting heart rate were measured in the dominant arm using an automated oscillometric device (Omron M300; Omron Healthcare; Kyoto, Japan). Three readings taken 1 min apart were averaged. We collected venous blood samples between 7:00 and 9:00 AM after a 12-h overnight fast. Serum glucose, total cholesterol (TC), high-density lipoprotein cholesterol (HDL-C), low-density lipoprotein cholesterol (LDL-C), and triglycerides (TG) were measured using a biochemical analyzer (BioChem SA). LDL-C was calculated using the Friedewald equation: LDL-C = TC − HDL-C − TG/2.2 (all in mmol/L).

### 2.2 Echocardiography

Echocardiography was performed by an ultrasound system (Affiniti 70, Philips Healthcare) with an S5-1 sector array transducer. All measurements followed American Society of Echocardiography/European Association of Cardiovascular Imaging (ASE/EACVI) chamber quantification guidelines [25]. Left atrial (LA) anteroposterior diameter was measured in the parasternal long-axis view at end-ventricular systole. LA volume (LAV) was calculated using the biplane area-length method from apical 4-chamber and 2-chamber views and indexed to BSA (LAVI). Interventricular septal thickness (IVS) and LV posterior wall (LVPW) thickness were obtained at end diastole immediately below the mitral valve leaflet tips from the parasternal long-axis view. LV end-diastolic (LVEDD) and end-systolic diameters (LVESD) were measured at the level of the mitral valve leaflet tips; corresponding volumes were derived using the modified Simpson biplane method from apical 4-chamber and 2-chamber views. Relative wall thickness (RWT) was computed as 2 × LVPW / LVEDD. LV mass was calculated using the linear formula: 0.8 × (1.04 × [(LVEDD + IVS + LVPW)^3^ − LVEDD^3^]) + 0.6 g and indexed to BSA [25]. LV ejection fraction was assessed using the Simpson biplane method. Transmitral early (E) and atrial (A) peak flow velocities were recorded using pulsed-wave Doppler with the sample volume placed at the mitral valve leaflet tips; the E/A ratio was then derived. Tissue Doppler early diastolic velocities (E′) were acquired at the septal and lateral mitral annulus; the average (E′ mean) was used to estimate the LV filling pressure (E/E′).

### 2.3 Statistical analysis

Data are presented as medians (interquartile range). Between-group differences were examined with Mann-Whitney *U* tests for continuous variables and Pearson χ^2^ tests for categorical variables. Partial Spearman rank correlations were used to assess associations of BMI, WC, and WHtR with the 13 echocardiographic parameters while accounting for age and systolic blood pressure (rank-residual method). False discovery rate (FDR) correction was applied using the Benjamini–Hochberg method jointly across all 39 adiposity-echocardiographic correlations. Approximate 95% confidence intervals for partial Spearman correlations were calculated using Fisher's z transformation. Cardiac parameters with significant FDR-corrected partial correlations with at least one adiposity measure were carried forward to multivariable linear regression adjusted for age, sex, and systolic blood pressure. Because BMI, WC, and WHtR are strongly correlated, each measure was entered in a separate model per outcome; standardized coefficients (Std β) and adjusted R^2^ are reported descriptively. Sensitivity analyses were performed by adding fasting triglycerides, LDL cholesterol, and fasting glucose to each primary regression model. Relative change in standardized β was calculated as (Std β metabolic-adjusted − Std β primary) / ǀStd β primaryǀ × 100. Sex-stratified echocardiographic characteristics are presented descriptively; to assess whether associations of WC with cardiac structural parameters differed by sex, a WC × sex interaction term was added to each regression model. Adjusted trends across WC quartiles were assessed using analysis of covariance (ANCOVA) models adjusted for age, sex, and systolic blood pressure, with quartile number entered as an ordinal predictor (β-trend, P-trend). Deviation from linearity was tested by F-test comparison of nested models with quartile as ordinal versus categorical predictors. Adjusted marginal means were estimated at mean age and mean systolic blood pressure with equal weighting of female and male participants. FDR-corrected P < 0.05 defined significance for the primary correlation analyses; nominal two-sided P values and 95% confidence intervals are reported descriptively for the regression, interaction, trend, and sensitivity analyses. All analyses were performed using R statistical software (version 4.5.1; R Core Team, R Foundation for Statistical Computing).

## 3 Results

A total of 100 participants (median age 39.0 years (30.8–49.2); 54% women) were enrolled. Clinical and metabolic characteristics are presented in Table 1. Participants with BMI ≥30 kg/m^2^ (n = 37) were significantly older than participants with BMI <30 kg/m^2^ (median 48.0 vs 34.0 years; P < 0.001), though sex distribution was comparable (P = 0.993). Forty-nine participants (49%) had elevated WC (WC quartile distribution: Q1 n = 23, Q2 n = 28, Q3 n = 25, Q4 n = 24); these participants were also significantly older than those with normal WC (median 49.0 vs 32.0 years; P < 0.001). Six participants (6% of the total cohort; 12.2% of those with elevated WC) had BMI (<25 kg/m^2^) with elevated WC (Supplementary Table 1). Participants with BMI ≥30 kg/m^2^ had higher blood pressure and fasting glucose, whereas participants with elevated WC had higher blood pressure, total and LDL cholesterol, triglycerides, and fasting glucose (Table 1).

**Table 1:** Clinical and metabolic characteristics of the study population stratified by BMI and waist circumference categories (n = 100).

Variable	All (n = 100)	BMI <30 kg/m^2^ (n = 63)	BMI ≥30 kg/m^2^ (n = 37)	P*	Normal WC (n = 51)	Elevated WC (n = 49)	P**
Sex (males/females)	46/54	29/34	17/20	0.993	26/25	20/29	0.308
Age (years)	39.0 (30.8–49.2)	34.0 (28.0–41.0)	48.0 (42.0–57.0)	**<0.001**	32.0 (26.0–37.5)	49.0 (42.0–57.0)	**<0.001**
Weight (kg)	76.0 (62.8–92.0)	65.0 (58.5–73.5)	94.0 (87.0–105.0)	**<0.001**	64.0 (57.0–72.5)	92.0 (80.0–101.0)	**<0.001**
BMI (kg/m^2^)	25.5 (23.2–33.0)	23.8 (22.1–24.8)	34.3 (32.8–38.7)	**<0.001**	23.2 (21.3–24.5)	33.1 (30.0–36.7)	**<0.001**
BSA (m^2^)	1.8 (1.7–2.0)	1.7 (1.6–1.9)	2.0 (1.9–2.2)	**<0.001**	1.7 (1.6–1.9)	2.0 (1.8–2.1)	**<0.001**
Waist (cm)	94.0 (82.0–107.0)	87.0 (78.2–92.8)	110.5 (105.0–117.0)	**<0.001**	82.0 (75.0–90.5)	107.0 (102.0–116.0)	**<0.001**
WHtR	0.56 (0.50–0.64)	0.51 (0.47–0.55)	0.67 (0.62–0.72)	**<0.001**	0.50 (0.45–0.54)	0.65 (0.61–0.71)	**<0.001**
SBP (mmHg)	115.0 (110.0–125.0)	110.0 (100.0–120.0)	122.5 (115.0–130.0)	**<0.001**	110.0 (100.0–120.0)	120.0 (110.0–130.0)	**<0.001**
DBP (mmHg)	78.5 (70.0–80.0)	70.0 (60.0–80.0)	80.0 (80.0–80.0)	**<0.001**	70.0 (60.0–80.0)	80.0 (70.0–80.0)	**<0.001**
LVEF (%)	58.5 (56.0–62.0)	59.0 (56.0–62.0)	57.5 (55.2–62.2)	0.206	60.0 (56.5–62.0)	58.0 (56.0–61.0)	0.051
Total cholesterol (mmol/L)	4.4 (3.9–5.0)	4.3 (3.9–4.9)	4.6 (3.9–5.0)	0.740	4.2 (3.9–4.7)	4.8 (3.9–5.1)	**0.019**
LDL cholesterol (mmol/L)	2.7 (2.2–3.3)	2.7 (2.1–3.3)	2.9 (2.3–3.4)	0.585	2.6 (2.1–3.1)	3.1 (2.3–3.8)	**0.028**
HDL cholesterol (mmol/L)	1.2 (1.1–1.3)	1.2 (1.1–1.4)	1.2 (1.1–1.3)	0.155	1.2 (1.1–1.4)	1.2 (1.1–1.3)	0.255
Triglycerides (mmol/L)	1.2 (1.0–1.4)	1.2 (0.9–1.3)	1.2 (1.1–1.6)	0.253	1.2 (0.9–1.3)	1.2 (1.1–1.7)	**0.035**
Blood glucose (mmol/L)	5.2 (4.6–5.5)	5.0 (4.5–5.3)	5.5 (5.1–5.8)	**<0.001**	5.0 (4.5–5.3)	5.4 (4.9–5.7)	**0.003**

Data are expressed as median (interquartile range) for continuous variables and counts for categorical variables. *P values correspond to body mass index (BMI) stratification; **P values correspond to waist circumference (WC) stratification. Both were obtained using the Mann-Whitney U test for continuous variables and the Pearson chi square test without continuity correction for categorical variables. Bold P-values indicate statistical significance (P < 0.05). BMI groups were defined as BMI <30 kg/m^2^ and BMI ≥30 kg/m^2^. Elevated WC was defined using sex-specific thresholds (men ≥102 cm; women ≥88 cm). Participants with WC < 102 cm (men) or <88 cm (women) were classified as having normal WC. Two-sided P < 0.05 was considered statistically significant. BSA, body surface area; DBP, diastolic blood pressure; HDL, high-density lipoprotein; LVEF, left ventricular ejection fraction; LDL, low-density lipoprotein; SBP, systolic blood pressure; WC, waist circumference; WHtR, waist-to-height ratio.

Echocardiographic characteristics stratified by sex are shown in Table 2. Males had significantly larger LA diameter (P = 0.003), IVS thickness (P = 0.007), LVEDD (P < 0.001), LVESD (P < 0.001), and LV mass (P < 0.001) than females. Anthropometric characteristics stratified by sex showed significant differences in weight, height, and WC between males and females (Supplementary Table 2). LVEF, BSA-indexed parameters (LAVI, LVMI), RWT and diastolic function indices did not differ significantly by sex (Table 2).

**Table 2:** Echocardiographic characteristics by sex (n = 100).

Parameter	Total (n = 100)	Males (n = 46)	Females (n = 54)	P-value
Left atrial parameters				
LA diameter (mm)	35.0 (32.0–37.2)	35.5 (34.0–38.8)	33.5 (31.0–36.8)	**0.003**
LA volume (mL)	52.0 (45.0–58.0)	55.0 (48.0–58.8)	51.0 (42.0–57.8)	0.093
LAVI (mL/m^2^)	28.1 (25.8–31.6)	27.3 (25.2–30.3)	28.9 (26.0–32.4)	0.058

LV wall thickness				
IVS thickness (mm)	9.0 (9.0–10.0)	9.5 (9.0–10.0)	9.0 (8.0–10.0)	**0.007**
LVPW thickness (mm)	9.0 (8.0–9.0)	9.0 (8.0–9.4)	8.2 (8.0–9.0)	0.077

LV chamber dimensions				
LVEDD (mm)	46.0 (44.0–49.0)	47.0 (45.2–50.0)	44.5 (42.0–47.0)	**<0.001**
LVESD (mm)	30.0 (27.0–32.0)	31.0 (29.0–33.0)	29.0 (26.2–30.0)	**<0.001**

LV mass				
LVM (g)	138.0 (118.0–162.5)	150.0 (137.0–164.0)	123.0 (104.5–153.0)	**<0.001**
LVMI (g/m^2^)	74.4 (67.1–83.8)	76.0 (68.2–88.0)	73.5 (65.0–80.5)	0.075

LV geometry				
RWT	0.38 (0.36–0.41)	0.37 (0.35–0.40)	0.39 (0.36–0.42)	0.144

Systolic function				
LVEF (%)	58.5 (56.0–62.0)	57.5 (55.2–61.0)	59.0 (57.0–63.5)	0.090

Diastolic function				
E/A ratio	1.30 (1.00–1.40)	1.30 (0.90–1.40)	1.30 (1.00–1.40)	0.979
E′ mean (cm/s)	10.8 (9.5–12.4)	10.8 (9.5–12.1)	10.8 (9.7–12.8)	0.727
E/E′ ratio	7.6 (6.8–8.4)	7.4 (6.5–8.2)	8.0 (6.9–8.8)	0.074

Data are median (interquartile range). P-values were derived from the Mann-Whitney U test. Available-case analysis was used for variables with missing measurements: E/A ratio and E/E′ ratio, n = 95; E′ mean, n = 98. All other parameters had n = 100. Bold P-values indicate statistical significance (P < 0.05). E/A, early-to-late diastolic transmitral velocity ratio; E/E′, early diastolic transmitral velocity to tissue Doppler E′ ratio; E′, early diastolic mitral annular velocity; IVS, interventricular septum; LA, left atrial; LAVI, left atrial volume index; LV, left ventricular; LVEDD, left ventricular end-diastolic diameter; LVEF, left ventricular ejection fraction; LVESD, left ventricular end-systolic diameter; LVM, left ventricular mass; LVMI, left ventricular mass index; LVPW, left ventricular posterior wall; RWT, relative wall thickness.

All three adiposity measures showed FDR-significant partial correlations with seven structural parameters: LA diameter, LA volume, IVS thickness, LVPW thickness, LVEDD, LVESD, and LV mass. For these seven parameters, partial correlation coefficients ranged from ρ = 0.332 to 0.602 for BMI, ρ = 0.443 to 0.636 for WC, and ρ = 0.314 to 0.549 for WHtR; corresponding 95% CIs are shown in Table 3 and Fig. 1. BSA-indexed parameters (LAVI, LVMI), RWT and diastolic function indices showed no FDR-significant adjusted associations.

**Table 3:** Partial Spearman rank correlations between echocardiographic parameters and adiposity measures, controlling for age and systolic blood pressure.

Parameter	ρ (BMI)	95% CI (BMI)	ρ (WC)	95% CI (WC)	ρ (WHtR)	95% CI (WHtR)
Left atrial parameters						
LA diameter (mm)	0.568***	0.417, 0.689	0.636***	0.500, 0.741	0.539***	0.382, 0.666
LA volume (mL)	0.602***	0.459, 0.715	0.598***	0.454, 0.712	0.549***	0.393, 0.674
LAVI (mL/m^2^)	0.115	−0.085, 0.307	0.005	−0.193, 0.203	0.144	−0.056, 0.333

LV wall thickness						
IVS thickness (mm)	0.345**	0.157, 0.508	0.525***	0.365, 0.655	0.434***	0.258, 0.582
LVPW thickness (mm)	0.370***	0.185, 0.529	0.502***	0.338, 0.637	0.425***	0.247, 0.575

LV chamber dimensions						
LVEDD (mm)	0.375***	0.191, 0.534	0.454***	0.281, 0.599	0.314**	0.124, 0.483
LVESD (mm)	0.332**	0.143, 0.498	0.443***	0.268, 0.589	0.323**	0.133, 0.490

LV mass						
LVM (g)	0.469***	0.298, 0.610	0.614***	0.473, 0.724	0.471***	0.301, 0.612
LVMI (g/m^2^)	0.042	−0.158, 0.238	0.163	−0.036, 0.351	0.136	−0.065, 0.325

LV geometry						
RWT	−0.038	−0.234, 0.162	−0.009	−0.207, 0.190	0.045	−0.155, 0.241

Diastolic function						
E/A ratio	−0.046	−0.248, 0.159	−0.100	−0.297, 0.106	−0.062	−0.262, 0.144
E′ mean (cm/s)	−0.067	−0.264, 0.135	−0.166	−0.355, 0.036	−0.077	−0.273, 0.126
E/E′ ratio	0.023	−0.182, 0.225	−0.047	−0.248, 0.159	0.024	−0.180, 0.227

Partial Spearman rank correlation coefficients (ρ) were computed via the rank-residual method, accounting for age and systolic blood pressure. Approximate 95% confidence intervals were calculated using Fisher's z transformation. Significance levels are FDR-corrected (Benjamini-Hochberg method across 39 tests): ***P < 0.001, **P < 0.01, *P < 0.05. Available-case analysis was used for parameters with missing measurements: E/A ratio and E/E′ ratio, n = 95; E′ mean, n = 98; all other parameters, n = 100. BMI, body mass index; E/A, early-to-late diastolic transmitral velocity ratio; E/E′, early diastolic transmitral velocity to tissue Doppler E′ ratio; E′, early diastolic mitral annular velocity; FDR, false discovery rate; IVS, interventricular septum; LA, left atrial; LAVI, left atrial volume index; LV, left ventricular; LVEDD, left ventricular end-diastolic diameter; LVESD, left ventricular end-systolic diameter; LVM, left ventricular mass; LVMI, left ventricular mass index; LVPW, left ventricular posterior wall; RWT, relative wall thickness; WC, waist circumference; WHtR, waist-to-height ratio; ρ, partial Spearman correlation coefficient.

**Fig. 1:**
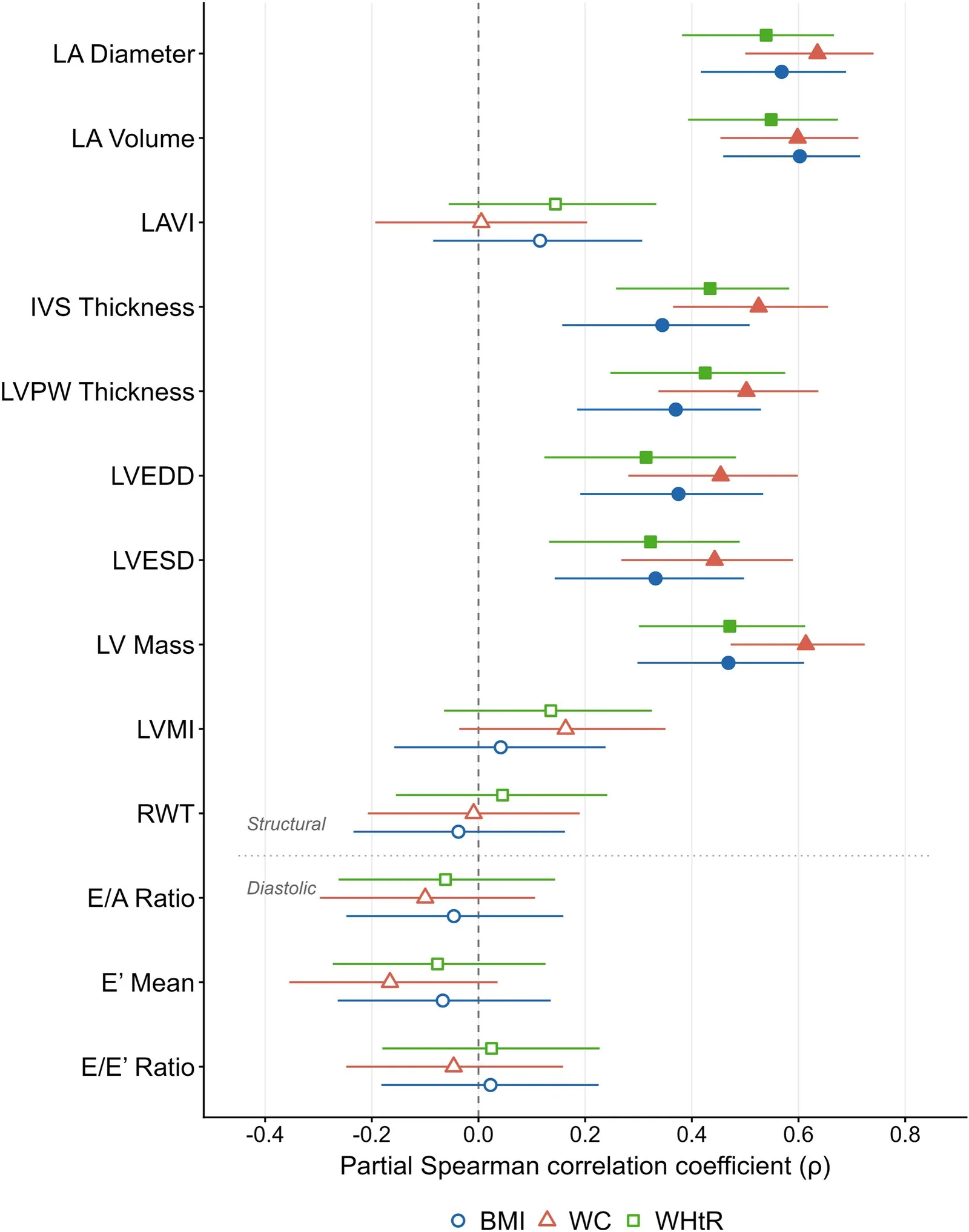
Partial Spearman correlations between adiposity measures and echocardiographic parameters. Partial Spearman correlation coefficients (ρ) were calculated using the rank-residual method, accounting for age and systolic blood pressure. Approximate 95% confidence intervals were calculated using Fisher's z transformation. P values were adjusted using the Benjamini–Hochberg procedure across all 39 correlations. Filled symbols indicate FDR-adjusted P < 0.05; open symbols indicate FDR-adjusted P ≥ 0.05. Available-case sample sizes were n = 100 for structural parameters, n = 95 for E/A ratio and E/E′ ratio, and n = 98 for E′ mean. BMI, body mass index; FDR, false discovery rate; IVS, interventricular septum; LA, left atrial; LAVI, left atrial volume index; LV, left ventricular; LVEDD, left ventricular end-diastolic diameter; LVESD, left ventricular end-systolic diameter; LVMI, left ventricular mass index; LVPW, left ventricular posterior wall; RWT, relative wall thickness; WC, waist circumference; WHtR, waist-to-height ratio.

Across the separate WC models, standardized β coefficients ranged from 0.375 to 0.582 (all P < 0.001) (Table 4 and Fig. 2). In sensitivity analyses additionally adjusting for triglycerides, LDL cholesterol, and fasting glucose, all 21 adiposity–cardiac structural associations remained nominally statistically significant. Relative changes in standardized β after additional metabolic adjustment ranged from −4.7% to +13.8% (Supplementary Table 4). No evidence of WC × sex interaction was observed for any parameter (all P-int > 0.05; Table 5). Adjusted trends across WC quartiles were observed for all structural parameters (all nominal P-trend < 0.05), with no evidence of deviation from linearity (all P-deviation > 0.05) (Table 6, Fig. 3). Adjusted LV mass increased from 127.67 g (95% CI, 114.80–140.54) in Q1 (62–81 cm) to 168.74 g (95% CI, 157.02–180.46) in Q4 (108–139 cm). Unadjusted scatter plots of WC against LV mass, LA volume, LA diameter, and LVEDD showed Spearman ρ of 0.75, 0.74, 0.75, and 0.51, respectively (Fig. 4). Applying the WHO Asian BMI action point of 27.5 kg/m^2^ reclassified 11 participants (7 men, 4 women) relative to the BMI ≥30 kg/m^2^ definition, increasing the number above the threshold from 37 to 48.

**Table 4:** Associations of adiposity measures with cardiac structural parameters in multivariable linear regression models adjusted for age, sex, and systolic blood pressure (n = 100).

Parameter	Adiposity	β (95% CI)	P	Std β	Std β 95% CI	Adj R^2^
Left atrial parameters						
LA diameter (mm)	BMI (per kg/m^2^)	0.302 (0.201 to 0.402)	<0.001	0.537	0.359 to 0.716	0.545
	WC (per cm)	0.133 (0.089 to 0.176)	<0.001	0.582	0.391 to 0.773	0.548
	WHtR (per 0.01)	0.209 (0.134 to 0.284)	<0.001	0.545	0.350 to 0.741	0.526
LA volume (mL)	BMI	1.104 (0.722 to 1.487)	<0.001	0.533	0.349 to 0.718	0.513
	WC	0.448 (0.278 to 0.619)	<0.001	0.534	0.331 to 0.737	0.490
	WHtR (per 0.01)	0.707 (0.415 to 1.000)	<0.001	0.500	0.294 to 0.707	0.472

LV wall thickness						
IVS thickness (mm)	BMI	0.047 (0.018 to 0.076)	0.002	0.275	0.106 to 0.443	0.596
	WC	0.026 (0.014 to 0.038)	<0.001	0.375	0.201 to 0.550	0.624
	WHtR (per 0.01)	0.042 (0.021 to 0.062)	<0.001	0.356	0.180 to 0.532	0.617
LVPW thickness (mm)	BMI	0.049 (0.020 to 0.078)	0.001	0.310	0.128 to 0.492	0.527
	WC	0.027 (0.015 to 0.039)	<0.001	0.415	0.226 to 0.604	0.558
	WHtR (per 0.01)	0.040 (0.019 to 0.061)	<0.001	0.370	0.177 to 0.563	0.540

LV chamber dimensions						
LVEDD (mm)	BMI	0.290 (0.161 to 0.418)	<0.001	0.467	0.259 to 0.674	0.386
	WC	0.122 (0.065 to 0.178)	<0.001	0.484	0.260 to 0.709	0.377
	WHtR (per 0.01)	0.152 (0.053 to 0.251)	0.003	0.358	0.124 to 0.592	0.323
LVESD (mm)	BMI	0.194 (0.089 to 0.300)	<0.001	0.402	0.184 to 0.621	0.319
	WC	0.085 (0.040 to 0.131)	<0.001	0.436	0.202 to 0.670	0.320
	WHtR (per 0.01)	0.109 (0.030 to 0.189)	0.008	0.332	0.090 to 0.573	0.279

LV mass						
LVM (g)	BMI	2.577 (1.620 to 3.535)	<0.001	0.446	0.280 to 0.612	0.608
	WC	1.190 (0.781 to 1.600)	<0.001	0.508	0.333 to 0.682	0.623
	WHtR (per 0.01)	1.699 (0.976 to 2.423)	<0.001	0.430	0.247 to 0.613	0.585

Separate multivariable linear regression models were constructed for each echocardiographic parameter. Each adiposity measure was entered individually, adjusted for age (years), sex, and systolic blood pressure (mmHg). WHtR coefficients represent the change per 0.01 unit (×100 rescaling). Adj R^2^, adjusted coefficient of determination; β, unstandardized regression coefficient; BMI, body mass index; CI, confidence interval; IVS, interventricular septum; LA, left atrial; LV, left ventricular; LVEDD, left ventricular end-diastolic diameter; LVESD, left ventricular end-systolic diameter; LVM, left ventricular mass; LVPW, left ventricular posterior wall; Std β, standardized regression coefficient; WC, waist circumference; WHtR, waist-to-height ratio.

**Fig. 2:**
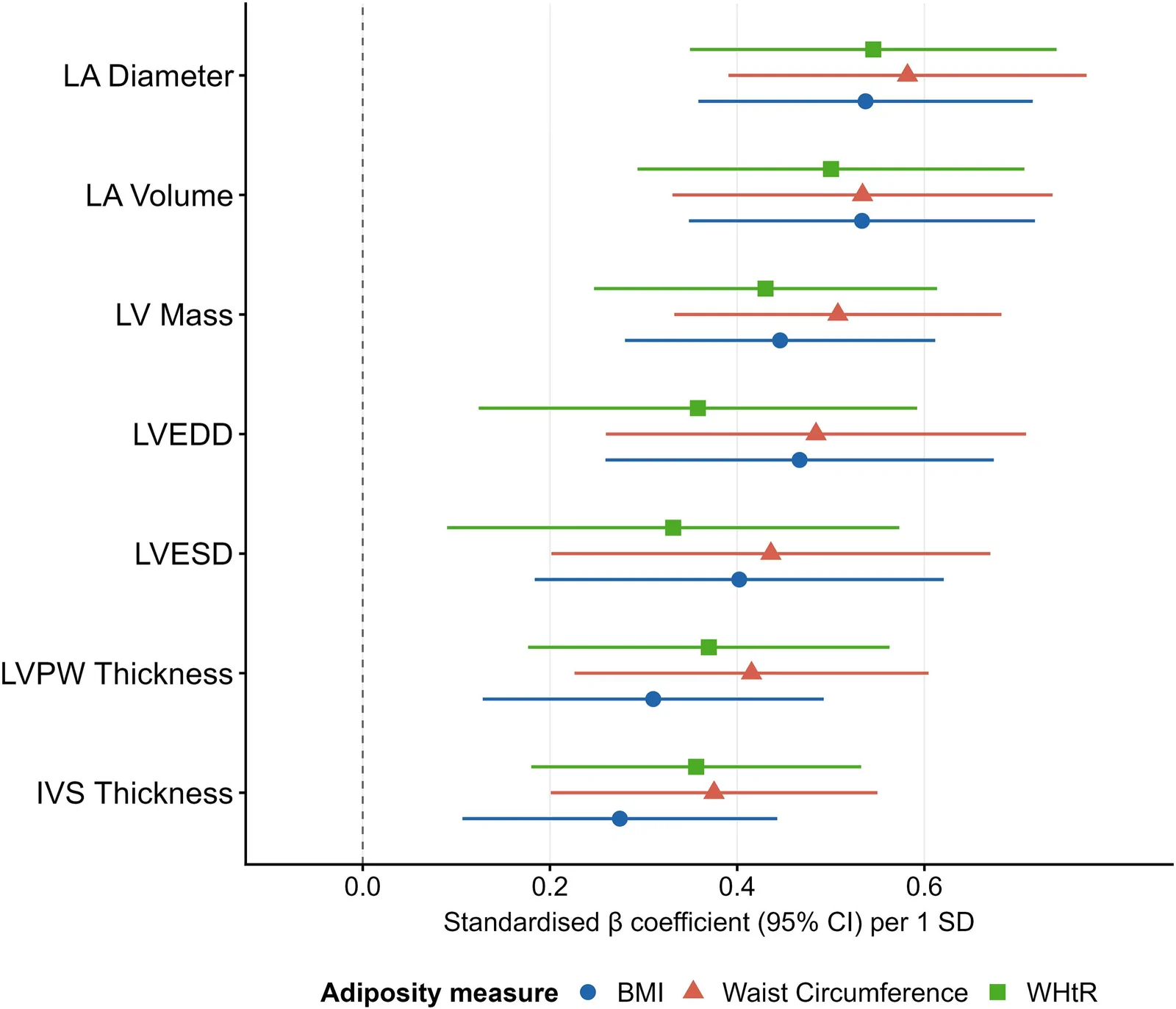
Standardized associations of adiposity measures with cardiac structural parameters. Standardized β coefficients and 95% confidence intervals are shown from separate multivariable linear regression models for BMI, WC, and WHtR, adjusted for age, sex, and systolic blood pressure (n = 100). BMI, body mass index; IVS, interventricular septum; LA, left atrial; LV, left ventricular; LVEDD, left ventricular end-diastolic diameter; LVESD, left ventricular end-systolic diameter; LVM, left ventricular mass; LVPW, left ventricular posterior wall; WC, waist circumference; WHtR, waist-to-height ratio.

**Table 5:** Waist circumference × sex interaction analysis (n = 100).

Parameter	WC coefficient in females (reference), β (95% CI)	P-WC	WC × sex interaction β (SE)	P-int
Left atrial parameters				
LA diameter (mm)	0.147 (0.098–0.196)	<0.001	−0.042 (0.034)	0.216
LA volume (mL)	0.418 (0.225–0.611)	<0.001	0.089 (0.133)	0.506

LV wall thickness				
IVS thickness (mm)	0.024 (0.010–0.038)	<0.001	0.006 (0.009)	0.517
LVPW thickness (mm)	0.027 (0.013–0.041)	<0.001	−0.001 (0.010)	0.953

LV chamber dimensions				
LVEDD (mm)	0.123 (0.059–0.188)	<0.001	−0.004 (0.044)	0.922
LVESD (mm)	0.089 (0.036–0.141)	<0.001	−0.009 (0.036)	0.800

LV mass				
LVM (g)	1.095 (0.632–1.558)	<0.001	0.280 (0.319)	0.382

Multivariable linear regression models included WC, sex (female = 0, male = 1), and the WC × sex interaction term as predictors, adjusted for age and systolic blood pressure. β, unstandardized regression coefficient; CI, confidence interval; Int, interaction; IVS, interventricular septum; LA, left atrial; LV, left ventricular; LVEDD, left ventricular end-diastolic diameter; LVESD, left ventricular end-systolic diameter; LVM, left ventricular mass; LVPW, left ventricular posterior wall; P-int, significance of the interaction term; P-WC, significance of the WC coefficient in females (reference); SE, standard error; WC, waist circumference

**Table 6:** Adjusted cardiac structural parameters across waist circumference quartiles (n = 100).

Parameter	Q1 (62–81 cm) n = 23	Q2 (82–94 cm) n = 28	Q3 (95–107 cm) n = 25	Q4 (108–139 cm) n = 24	β-Trend	P-trend	P-deviation
Left atrial parameters							
LA diameter (mm)	32.59 (31.26–33.92)	32.96 (31.96–33.96)	36.07 (35.00–37.15)	37.31 (36.10–38.53)	1.782	<0.001	0.101
LA volume (mL)	48.28 (42.89–53.67)	49.45 (45.40–53.50)	56.08 (51.74–60.42)	63.08 (58.17–67.99)	5.319	<0.001	0.351

LV wall thickness							
IVS thickness (mm)	9.10 (8.73–9.48)	9.16 (8.88–9.44)	9.49 (9.19–9.79)	10.02 (9.68–10.36)	0.326	<0.001	0.295
LVPW thickness (mm)	8.27 (7.89–8.65)	8.48 (8.20–8.77)	8.80 (8.49–9.11)	9.28 (8.93–9.63)	0.343	<0.001	0.682

LV chamber dimensions							
LVEDD (mm)	44.25 (42.48–46.02)	45.01 (43.68–46.34)	46.44 (45.01–47.86)	48.53 (46.92–50.14)	1.469	0.001	0.652
LVESD (mm)	28.88 (27.44–30.31)	28.69 (27.61–29.76)	30.15 (28.99–31.30)	31.20 (29.90–32.51)	0.896	0.012	0.393

LV mass							
LVM (g)	127.67 (114.80–140.54)	130.53 (120.85–140.20)	143.39 (133.02–153.77)	168.74 (157.02–180.46)	14.313	<0.001	0.103

Values are adjusted marginal means (95% CI) by WC quartile, adjusted for age, sex, and systolic blood pressure. Marginal means were estimated at mean age and mean systolic blood pressure with equal weighting of female and male participants. β-Trend was estimated from an ordinal WC-quartile model and represents the change per one-quartile increase. P-deviation from linearity tests whether modelling WC quartile categorically improves model fit compared with the ordinal linear-trend model. β, unstandardized regression coefficient; CI, confidence interval; IVS, interventricular septum; LA, left atrial; LV, left ventricular; LVEDD, left ventricular end-diastolic diameter; LVESD, left ventricular end-systolic diameter; LVM, left ventricular mass; LVPW, left ventricular posterior wall; P-deviation, P-value for deviation from linearity; P-trend, P-value for linear trend; Q, quartile; WC, waist circumference.

**Fig. 3:**
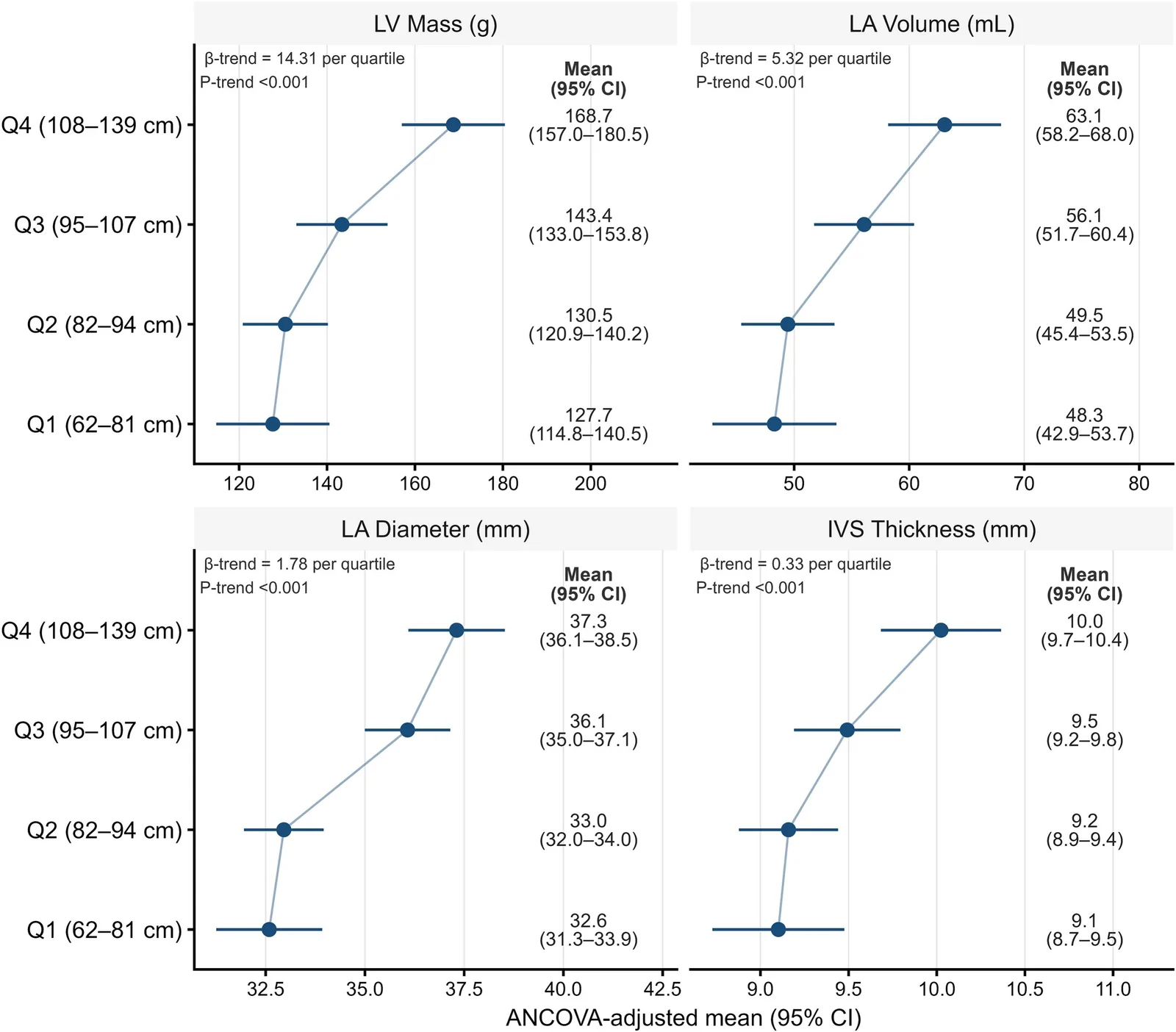
Adjusted cardiac structural parameters across waist circumference quartiles. Adjusted marginal means and 95% confidence intervals are shown across observed WC quartiles: Q1, 62–81 cm (n = 23); Q2, 82–94 cm (n = 28); Q3, 95–107 cm (n = 25); and Q4, 108–139 cm (n = 24). Models were adjusted for age, sex, and systolic blood pressure, with marginal means estimated at mean age and mean systolic blood pressure and equal weighting of female and male participants. P values for linear trend and deviation from linearity are reported in Table 6. ANCOVA, analysis of covariance; IVS, interventricular septum; LA, left atrial; LVM, left ventricular mass; Q, quartile; WC, waist circumference.

**Fig. 4:**
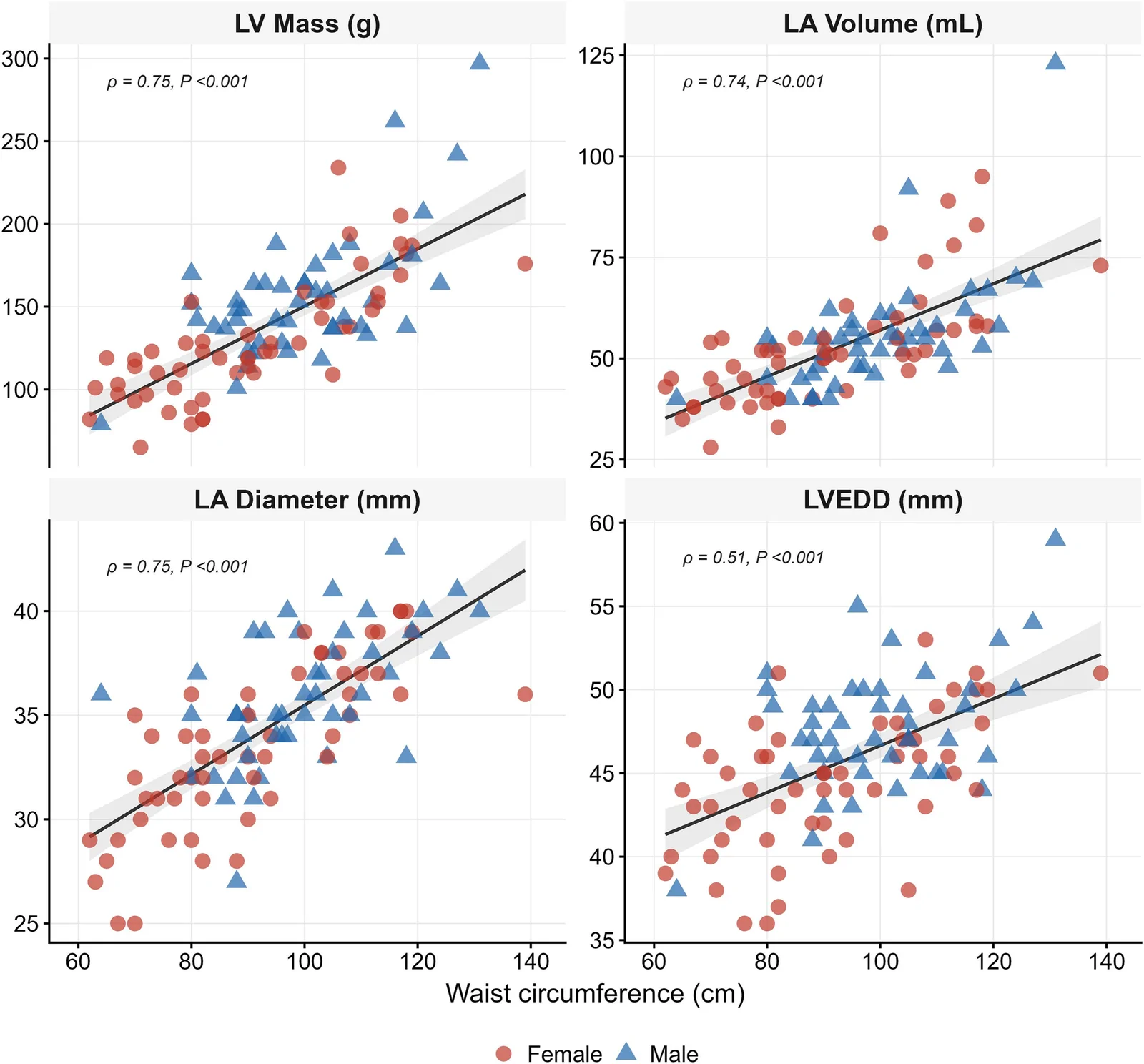
Unadjusted associations between waist circumference and selected cardiac structural parameters. Scatterplots show unadjusted relationships between waist circumference and left ventricular mass, left atrial volume, left atrial diameter, and left ventricular end-diastolic diameter, each with a pooled linear fit (shaded 95% CI) and the unadjusted Spearman correlation coefficient (ρ = 0.75, 0.74, 0.75, and 0.51, respectively; all P < 0.001; n = 100). Data points are colored and shaped by sex for display. ρ, Spearman rank correlation coefficient; LA, left atrial; LVEDD, left ventricular end-diastolic diameter; LVM, left ventricular mass; WC, waist circumference.

## 4 Discussion

In this study of Kyrgyz adults without established cardiovascular disease, we compared associations of general (BMI) and central (WC, WHtR) adiposity measures with cardiac structural and functional parameters. Greater adiposity was associated with several cardiac structural measures, while systolic function was preserved at the cohort level. WC showed positive adjusted associations with structural measures, as did BMI and WHtR for the same seven structural parameters. These findings highlight associations between adiposity, including central adiposity, and cardiac structure in this cohort.

### 4.1 Cardiac remodeling phenotype

IVS thickness, LVPW thickness, and LV mass showed FDR-significant positive partial correlations with BMI, WC, and WHtR (Table 3). The ASE/EACVI 2015 guidelines define LV hypertrophy at LVMI >115 g/m^2^ in men and >95 g/m^2^ in women [25]; higher LV mass and LVMI have been associated with increased cardiovascular risk in population studies [26], [27], [28], [29]. RWT showed no FDR-significant adjusted associations with adiposity (Table 3); together with the wall-thickness findings, this describes structural remodeling without a consistent concentric or eccentric geometric pattern. Recent meta-analytic data show predominant eccentric remodeling in obesity (66% vs 34% concentric pattern) [30]. The observed associations with absolute wall thickness, without a corresponding FDR-significant association with RWT, may reflect differences in obesity severity, duration, and comorbidity burden. Eccentric remodeling typically develops from volume overload secondary to expanded blood volume perfusing increased adipose tissue mass [31], [32], whereas concentric remodeling occurs when arterial hypertension imposes pressure overload [33], [34]. Our sample exhibited a median BMI 34.3 kg/m^2^ among participants with BMI ≥30 kg/m^2^, higher blood pressure values, and absence of established cardiovascular disease; these factors may contribute to the wall thickening observed without definitive concentric geometry. Evidence suggests adverse cardiac remodeling may become evident at lower BMI thresholds, with values as low as 23–24 kg/m^2^ [35], [36], [37], consistent with associations between BMI and structural cardiac measures observed in this cohort.

### 4.2 Central and general adiposity and cardiac alterations

WC was positively associated with several cardiac structural parameters after adjustment for age and systolic blood pressure. WC is a practical anthropometric measure of central adiposity and an indirect marker of visceral adiposity. Visceral adipose tissue exhibits increased lipolytic activity, increased proinflammatory cytokine secretion (TNF-α, IL-6), and greater contribution to systemic insulin resistance [38]. Prior studies link visceral adiposity with cardiac remodeling through inflammatory, metabolic, neurohormonal, and lipotoxic pathways [32], [38]. Across the separate adjusted models, BMI, WC, and WHtR were each associated with the seven structural outcomes identified in the partial-correlation analysis (Table 4). The findings are broadly consistent with prior evidence demonstrating clinical and prognostic relevance of central adiposity [16], [20], [39], [40]. Recently, phenotyped groups of individuals with normal weight but central obesity (NWCO) have been shown to carry a higher cardiovascular risk than those with general obesity. For example, among postmenopausal women, NWCO was associated with increased all-cause and cardiovascular mortality compared to normal weight without central obesity [41]. Moreover, a recent retrospective analysis showed elevated risks of heart failure, atrial fibrillation, and angina in NWCO [42]. NWCO individuals exhibited higher mortality than individuals with overweight or obesity classified by BMI alone [43]. In our Kyrgyz cohort, 6% of participants had BMI <25 kg/m^2^ with elevated WC (Supplementary Table 1). This discordance between BMI- and WC-defined adiposity appears particularly pronounced in Kyrgyz populations: a regional survey in rural Chui province found 52.3% central obesity by waist circumference despite a BMI-defined obesity prevalence of only 25.7% [44], consistent with evidence that populations across Central Asia, the Middle East, and North Africa reach equivalent waist-to-height ratios at systematically lower BMI than Western populations [45]. This regional burden is compounded by a substantially elevated cardiovascular disease burden: Central Asia carries the highest age-standardized cardiovascular disability-adjusted life-year rate among six Asian regions [46], alongside the 2.4-fold elevated ischemic heart disease mortality already noted [23].

### 4.3 Left atrial remodeling

LA structural measures showed positive partial correlations with WC (ρ = 0.598 for LA volume and ρ = 0.636 for LA diameter). Ten participants (10%) had LAVI >34 mL/m^2^, the ASE/EACVI (2015) upper reference value used to classify LA enlargement [25] (Supplementary Table 3); adjusted LA volume was higher across WC quartiles. The association between WC and LA structural parameters, after adjustment for age and systolic blood pressure, supports an association between central adiposity and LA structure in this cohort. LA enlargement is clinically relevant because of the established association between LA enlargement and atrial fibrillation [47], [48], which shows higher prevalence in obesity. These findings indicate larger adjusted LA and LV structural measures across the WC distribution in adults without established cardiovascular disease.

### 4.4 Structural remodeling and measured diastolic indices

Preserved LV ejection fraction coexisted with structural cardiac remodeling in this cohort. The measured diastolic indices showed no FDR-significant adjusted associations with adiposity (Table 3). Thus, the cross-sectional findings characterize structural abnormalities alongside preserved systolic function and an absence of statistically significant adjusted associations with the measured diastolic indices. Overall, 32% of participants had at least one structural measurement exceeding an ASE/EACVI reference threshold (Supplementary Table 3). Because echocardiographic reference values differ across ethnicities [49] and are unavailable for Kyrgyz adults, this threshold-based prevalence should be interpreted descriptively.

## 5 Study limitations

This study has several limitations. First, the substantial age differences between participants with BMI ≥30 kg/m^2^ and BMI <30 kg/m^2^, and between those with elevated WC and normal WC, represent potential confounders. Although age was included in the adjusted models, residual confounding by unmeasured age-related factors may remain. Second, the modest sample size (n = 100), single-center, urban recruitment, and cross-sectional design constrain generalizability. Third, our cohort represents a selected population without established cardiovascular disease, diabetes, or hypertension, which limits generalizability to unselected populations or those with prevalent comorbidities. The modest sample size limits precision, particularly for small or nonsignificant associations; the absence of statistical significance for the evaluated diastolic parameters should not be interpreted as evidence of no association. The use of a BMI threshold of 30 kg/m^2^ for the primary obesity classification may misclassify adiposity in Asian populations. The WHO expert consultation identified additional BMI action points for Asian populations, including 23 and 27.5 kg/m^2^ [50], while population-specific thresholds for Central Asian populations, including the Kyrgyz population, remain uncertain. Moreover, we did not assess other measures of central adiposity, including WHR and directly measured VAT. Comprehensive metabolic phenotyping, including oral glucose tolerance testing and direct assessment of insulin resistance, was not performed, which limits mechanistic interpretation of the observed adiposity-cardiac associations.

## 6 Conclusions

In Kyrgyz adults without established cardiovascular disease, WC showed adjusted associations with cardiac structural measures, while BMI and WHtR were also associated with the same seven structural parameters. Adjusted trends were observed across WC quartiles, with no evidence of WC × sex interaction. Structural remodeling coexisted with preserved systolic function and no FDR-significant adjusted associations for the measured diastolic indices. Large prospective studies incorporating direct visceral-fat assessment and longitudinal follow-up can further define the clinical relevance of WC-associated cardiac remodeling.

## CRediT authorship contribution statement

**Kylychbek Suiunov:** Methodology, Investigation, Formal analysis, Data curation, Conceptualization, Writing – original draft, Writing – review & editing. **Andrey Polupanov:** Supervision, Methodology, Investigation, Formal analysis, Data curation, Conceptualization, Writing – original draft, Writing – review & editing. **Kyialbek Sakibaev:** Methodology, Investigation, Formal analysis, Data curation, Writing – review & editing. **Meerimgul Sherikbai kyzy:** Methodology, Investigation, Formal analysis, Data curation, Writing – review & editing. **Zhazgul Imetova:** Methodology, Investigation, Formal analysis, Data curation, Writing – review & editing. **Anara Zhanbaeva:** Methodology, Investigation, Formal analysis, Data curation, Writing – review & editing. **Nazgul Tashmatova:** Methodology, Investigation, Formal analysis, Data curation, Writing – review & editing. **Aigul Momunova:** Methodology, Investigation, Formal analysis, Data curation, Writing – review & editing. **Lazokatkhan Dzhumaeva:** Methodology, Investigation, Formal analysis, Data curation, Writing – review & editing. **Akpay S. Sarybaev:** Methodology, Investigation, Formal analysis, Data curation, Writing – review & editing. **Akylbek Sydykov:** Methodology, Investigation, Formal analysis, Data curation, Writing – original draft, Writing – review & editing. **Argen Mamazhakypov:** Visualization, Supervision, Project administration, Methodology, Investigation, Formal analysis, Data curation, Conceptualization, Writing – original draft, Writing – review & editing.

## Informed consent

Written informed consent was obtained from all participants prior to inclusion in the study.

## Ethics committee approval

The study was approved by the Institutional Ethical Committee of Osh State University, Osh, Kyrgyzstan (Approval No.:13, approved on 29 June 2024).

## Funding

This study received no external funding.

## Declaration of competing interest

The authors have no conflicts of interest to declare.
