# Supplementary material for: Central adiposity and subclinical cardiac remodeling in Kyrgyz adults
Source: Am Heart J Plus. 2026 Oct 24;72:100992. doi: 10.1016/j.ahjo.2026.100992 (PMC13755458; doi:10.1016/j.ahjo.2026.100992)
Supplement: Supplementary file 1 [file mmc1.docx]

Supplementary Table 1. BMI categories and combined adiposity classification by sex (n=100)

| Category | Total (n=100) | Males (n=46) | Females (n=54) | P value |
| --- | --- | --- | --- | --- |
| *BMI Categories* |  |  |  | 0.677 |
| Underweight (< 18.5 kg/m^2^) | 3 (3.0) | 1 (2.2) | 2 (3.7) |  |
| Normal weight (18.5 - 24.9 kg/m^2^) | 47 (47.0) | 20 (43.5) | 27 (50) |  |
| Overweight (25.0 - 29.9 kg/m^2^) | 13 (13.0) | 8 (17.4) | 5 (9.3) |  |
| Obesity (≥ 30.0 kg/m^2^) | 37 (37.0) | 17 (37.0) | 20 (37.0) |  |
| *Waist-based adiposity measures* |  |  |  | 0.308 |
| Normal WC* | 51 (51.0) | 26 (56.5) | 25 (46.3) |  |
| Elevated WC* | 49 (49.0) | 20 (43.5) | 29 (53.7) |  |
| *Combined adiposity*** |  |  |  | 0.074 |
| BMI < 25 kg/m^2^ without elevated WC | 44 (44.0) | 20 (43.5) | 24 (44.4) |  |
| BMI < 25 kg/m^2^ with elevated WC | 6 (6.0) | 1 (2.2) | 5 (9.3) |  |
| BMI ≥ 25 kg/m^2^ without elevated WC | 7 (7.0) | 6 (13.0) | 1 (1.9) |  |
| BMI ≥ 25 kg/m^2^ with elevated WC | 43 (43.0) | 19 (41.3) | 24 (44.4) |  |

Data are presented as number (percentage) with percentages calculated within each sex group using sex-specific denominators (males, n = 46; females, n = 54). Total column percentages were calculated using the entire study population as the denominator (N=100). Body mass index (BMI) was categorized as underweight (< 18.5 kg/m^2^), normal weight (18.5 - 24.9 kg/m^2^), overweight (25.0-29.9 kg/m^2^), and obesity (≥ 30.0 kg/m^2^). *Elevated waist circumference (WC) was defined using sex-specific WC thresholds: ≥102 cm for males and ≥88 cm for females. **Combined adiposity was derived by cross-classifying BMI (< 25.0 vs ≥ 25.0 kg/m^2^) with WC status. P values compare males versus females using Pearson's χ^2^ test of independence; Monte Carlo simulation (2000 replicates) was employed when expected cell frequencies were <5.

Supplementary Table 2. Complete anthropometric and obesity profile by sex (n = 100)

| Category | Total (n=100) | Males (n=46) | Females (n=54) | P |
| --- | --- | --- | --- | --- |
| Weight (kg) | 76.0 (62.8–92.0) | 82.0 (72.0–94.0) | 66.0 (58.2–86.0) | <0.001 |
| Height (cm) | 165.0 (160.0–173.2) | 173.5 (170.0–178.0) | 160.0 (157.0–163.0) | <0.001 |
| BSA (m^2^) | 1.8 (1.7–2.0) | 2.0 (1.9–2.1) | 1.7 (1.6–1.9) | <0.001 |
| BMI (kg/m^2^) | 25.5 (23.2–33.0) | 28.2 (23.9–32.3) | 24.4 (22.8–34.4) | 0.686 |
| Waist (cm) | 94.0 (82.0–107.0) | 98.0 (90.0–107.8) | 90.0 (77.2–105.8) | 0.009 |
| WHtR | 0.56 (0.50–0.64) | 0.56 (0.52–0.63) | 0.55 (0.49–0.66) | 0.470 |
|  |  |  |  |  |
| Obesity (≥ 30.0 kg/m^2^) | 37 (37.0) | 17 (37.0) | 20 (37.0) | 0.993 |
| Elevated WC* | 49 (49.0) | 20 (43.5) | 29 (53.7) | 0.308 |

Data are expressed as median (interquartile range) for continuous variables or as number (percentage (%)) for categorical variables. Percentages were calculated using sex-specific denominators. P values compare males versus females using the Mann-Whitney U test for continuous variables and the Pearson chi-square test without continuity correction for categorical variables. Waist-to-height ratio (WHtR) = waist circumference (cm)/height (cm). *Elevated WC was defined using sex-specific waist circumference thresholds (men ≥ 102 cm; women ≥ 88 cm). BMI, body mass index; BSA, body surface area.

Supplementary Table 3. Prevalence of echocardiographic measurements exceeding ASE/EACVI reference thresholds (n = 100)

| Threshold above normal | Total (n = 100) (n (%)) | Males (n = 46) (n (%)) | Females (n = 54) (n (%)) | P** |
| --- | --- | --- | --- | --- |
| LA diameter > 40 mm (men) or > 38 mm (women) | 10 (10.0) | 3 (6.5) | 7 (13.0) | 0.33 |
| LAVI > 34 mL/m^2^ | 10 (10.0) | 3 (6.5) | 7 (13.0) | 0.33 |
| IVS or LVPW > 10 mm (men) or > 9 mm (women) | 29 (29.0) | 9 (19.6) | 20 (37.0) | 0.077 |
| LVMI > 115 g/m^2^ (men) or > 95 g/m^2^ (women) | 4 (4.0) | 2 (4.3) | 2 (3.7) | 1.00 |
| LVEDD >58 (men) or > 52 (women) | 2 (2.0) | 1 (2.2) | 1 (1.9) | 1.00 |
| Any structural threshold exceedance* | 32 (32.0) | 9 (19.6) | 23 (42.6) | 0.018 |

*Defined as the presence of one or more of the following: LA diameter > 40 mm (men) or > 38 mm (women), LAVI > 34 mL/m^2^, IVS or LVPW > 10 mm (men) or > 9 mm (women), LVMI > 115 g/m^2^ (men) or > 95 g/m^2^ (women), or LVEDD > 58 mm (men) or > 52 mm (women). ** Two-sided Fisher’s exact test was used to compare males and females. ASE/EACVI (2015) guideline thresholds were applied. ASE, American Society of Echocardiography; EACVI, European Association of Cardiovascular Imaging; IVS, interventricular septum; LAVI, left atrial volume index; LV, left ventricular; LVEDD, left ventricular end-diastolic diameter; LVMI, left ventricular mass index; LVPW, left ventricular posterior wall.

Supplementary Table 4. Sensitivity analysis of associations between adiposity measures and cardiac structural parameters in multivariable linear regression models with additional adjustment for triglycerides, LDL cholesterol, and fasting glucose (n = 100)

| Parameter | Adiposity | Std β (primary model) | Std β (metabolic-adjusted) | Relative change (%) | P | Adj R^2^ |
| --- | --- | --- | --- | --- | --- | --- |
| *Left atrial parameters* |  |  |  |  |  |  |
| LA diameter (mm) | BMI (per kg/m^2^) | 0.537 | 0.527 | - 2.0 | <0.001 | 0.554 |
|  | WC (per cm) | 0.582 | 0.560 | -3.8 | <0.001 | 0.548 |
|  | WHtR (per 0.01) | 0.545 | 0.528 | -3.1 | <0.001 | 0.526 |
| LA volume (mL) | BMI | 0.533 | 0.534 | 0.2 | <0.001 | 0.500 |
|  | WC | 0.534 | 0.543 | 1.7 | <0.001 | 0.479 |
|  | WHtR (per 0.01) | 0.500 | 0.530 | 6.0 | <0.001 | 0.467 |
| *LV wall thickness* |  |  |  |  |  |  |
| IVS thickness (mm) | BMI | 0.275 | 0.268 | -2.6 | 0.002 | 0.595 |
|  | WC | 0.375 | 0.361 | - 3.9 | <0.001 | 0.619 |
|  | WHtR (per 0.01) | 0.356 | 0.339 | - 4.7 | <0.001 | 0.609 |
| LVPW thickness (mm) | BMI | 0.310 | 0.305 | -1.8 | 0.001 | 0.528 |
|  | WC | 0.415 | 0.405 | -2.5 | <0.001 | 0.556 |
|  | WHtR (per 0.01) | 0.370 | 0.363 | -1.8 | <0.001 | 0.538 |
| *LV chamber dimensions* |  |  |  |  |  |  |
| LVEDD (mm) | BMI | 0.467 | 0.471 | 1.0 | <0.001 | 0.404 |
|  | WC | 0.484 | 0.504 | 4.1 | <0.001 | 0.400 |
|  | WHtR (per 0.01) | 0.358 | 0.400 | 11.7 | 0.001 | 0.349 |
| LVESD (mm) | BMI | 0.402 | 0.406 | 1.1 | <0.001 | 0.346 |
|  | WC | 0.436 | 0.454 | 4.1 | <0.001 | 0.352 |
|  | WHtR (per 0.01) | 0.332 | 0.377 | 13.8 | 0.003 | 0.317 |
| *LV mass* |  |  |  |  |  |  |
| LVM (g) | BMI | 0.446 | 0.446 | 0.0 | <0.001 | 0.598 |
|  | WC | 0.508 | 0.515 | 1.4 | <0.001 | 0.614 |
|  | WHtR (per 0.01) | 0.430 | 0.451 | 4.7 | <0.001 | 0.578 |

Std β for the primary model was adjusted for age, sex, and systolic blood pressure. The metabolic-adjusted models additionally included triglycerides, LDL cholesterol, and fasting glucose; all variables were available for all 100 participants. Relative change in standardized β (%) was calculated as (Std β metabolic-adjusted − Std β primary)/|Std β primary| × 100. P values and adjusted R^2^ values shown are from the metabolic-adjusted models. Adj R^2^, adjusted coefficient of determination; BMI, body mass index; IVS, interventricular septum; LA, left atrial; LDL, low-density lipoprotein; LV, left ventricular; LVEDD, left ventricular end-diastolic diameter; LVESD, left ventricular end-systolic diameter; LVM, left ventricular mass; LVPW, left ventricular posterior wall; P, P value for the metabolic-adjusted model; Std β, standardized regression coefficient; WC, waist circumference; WHtR, waist-to-height ratio.
